# Effect of Cooking Methods on Bioactivity of Polyphenols in Purple Potatoes

**DOI:** 10.3390/antiox10081176

**Published:** 2021-07-24

**Authors:** Qi Sun, Min Du, Duroy A. Navarre, Meijun Zhu

**Affiliations:** 1 School of Food Science, Washington State University, Pullman, WA 99164, USA; qi.sun6@wsu.edu; 2 Department of Animal Science, Washington State University, Pullman, WA 99164, USA; min.du@wsu.edu; 3 Temperate Tree Fruit and Vegetable Research Unit, USDA-Agricultural Research Service, Prosser, WA 99350, USA; roy.navarre@usda.gov

**Keywords:** purple potato, chlorogenic acid, polyphenol, antioxidant, anti-inflammatory, cooking, Caco-2 cells

## Abstract

Purple-fleshed potato (*Solanum tuberosum* L.) is a good dietary source of anthocyanins, flavonols, and polyphenolic acids, mostly chlorogenic acid. The objective of this study was to examine the impacts of cooking methods including boiling, steaming, and the newly developed vacuum-sealed boiling (VSBoil) on extractability and bioactivity of polyphenolic compounds in a purple potato (PP) cultivar, Purple Pelisse. Data showed that boiling and steaming reduced the total polyphenolic content in PP. High-performance liquid chromatography analysis showed that steaming slightly reduced the extractable chlorogenic acid content, while VSBoil increased it. For DPPH free radical scavenging activities, VSBoil and steaming effectively preserved the antioxidant activity of a polyphenol-rich extract of PP, while boiling resulted in a significant reduction compared to raw potato extract. All extracts effectively suppressed bursts of intracellular reactive oxygen species in human colonic epithelial cells upon hydrogen peroxide induction, of which the extract from the VSBoil group showed the highest antioxidant potential. In addition, all extracts showed anti-inflammatory effects in Caco-2 cells induced with tumor necrosis factor-α. In conclusion, the content and bioactivity of extractable polyphenols were largely retained in PP subjected to different cooking processes. VSBoil resulted in the highest content of extractable polyphenolic compounds and bioactivity among tested cooking methods.

## 1. Introduction

Potatoes, the carbohydrate-rich tubers of *Solanum tuberosum* L., are widely grown and consumed around the world. As a staple food, potato is a good source of natural phytochemicals. In addition to macronutrients, potato tubers are enriched in phytonutrients such as dietary fibers and polyphenols [1]. Pigmented potato tubers, such as purple-fleshed potatoes, contain anthocyanins that are responsible for their red, blue, or purple color. Purple potato (PP) is also rich in polyphenolic acids, including chlorogenic acid (CGA) and caffeic acid, which are higher than those in white potato [2]. Purple Pelisse, the variety we used in this study, was first cross bred in 2000 and yielded purple skin and dark purple flesh tubers [3].

The beneficial effects of PP and its extracts have been examined both in vitro and in vivo. PP supplementation enhanced the activities of antioxidant enzymes, including catalase, glutathione *S*-transferase, glutathione reductase [4], and reduced serum lipid profile/cholesterol level [5] in cholesterol-fed rats. In streptozotocin-induced diabetic rats, supplementation of PP extract improved the activities of antioxidant enzymes in leukocytes and reduced malondialdehyde content in blood plasma [6]. Meanwhile, PP’s anti-hepatotoxicity [7], anti-inflammatory [8] and anti-cancer effect [9], as well as promoting effects on intestinal epithelial differentiation [10] were also discovered.

Potatoes serve as raw materials of side dishes, snacks, brewing and edible starches, and are always cooked before consumption. However, most available studies used uncooked potatoes or polyphenol-rich extract from uncooked potatoes as experimental materials for dietary supplementation and mouse feeding studies, which are not consistent with human eating habits. Thus, it is important to evaluate the impact of different cooking methods on phytochemical content and bioavailability in PP. Cell-free chemical analyses indicated that cooking processes such as boiling, baking, or microwaving reduced total polyphenolic content and the in vitro antioxidant activities of PP [11]. The antioxidant capacity and anti-inflammatory activities of PP subjected to different cooking processes have not been directly tested in live-cell systems.

In the present study, we tested a new cooking method, vacuum-sealed boiling (VSBoil, also known as vacuum cooking), where potatoes are first vacuum sealed and then boiled in water, along with boiling (cooked directly in boiling water) and steaming (cooked in steam generated by boiling water). We assessed the impacts of these three cooking methods on the content, profile, antioxidant activity of polyphenols in PP. The antioxidant and anti-inflammatory effects of the polyphenol extracts of PP were further examined in human colonic epithelial Caco-2 cells.

## 2. Materials and Methods

### 2.1. Chemicals and Materials

Normal hexane (*n*-hexane), methanol, formic acid, thiazolyl blue tetrazolium bromide (MTT), methyl sulfoxide (DMSO), 2,2-diphenyl-1-picrylhydrazyl (DPPH), ethanol, CGA, gallic acid (GA), and 2,7-dichlorofluorescein diacetate (DCFH-DA) were purchased from Sigma (St. Louis, MO, USA). Folin–Ciocalteu’s reagent was purchased from MP Biomedicals (Irvine, CA, USA). Sodium carbonate (Na_2_CO_3_) was purchased from JT Baker Chemicals (Phillipsburg, NJ, USA). Hydrogen peroxide (H_2_O_2_) was obtained from Honeywell (Charlotte, NC, USA). HPLC grade *n*-hexane, methanol, and formic acid were used. All other chemicals were of analytical grade.

The human colonic epithelial cell line Caco-2 was obtained from the American Type Culture Collection (Manassas, VA, USA). Caco-2 cells were routinely cultured in Dulbecco’s modified Eagle’s medium (DMEM) complete media: DMEM (Sigma, St. Louis, MO, USA) supplemented with 10% fetal bovine serum (Sigma), 100 units/mL penicillin G, and 100 μg/mL of streptomycin (Sigma) at 37 °C with 5% CO_2_. Recombinant human tumor necrosis factor (TNF)-α was purchased from Cell Signaling Technology (Beverly, MA, USA).

### 2.2. Polyphenol-Rich Purple Potato Extract Preparation

Fresh Purple Pelisse PP was grown and harvested in the Pacific Northwest. Potatoes with an average diameter of 4.3 ± 0.3 cm were randomly divided into four groups. In boiling (Boil) and steaming (Steam) treatments, potatoes (unpeeled) were submerged in boiled water (~2.5 L) or steam cooked for 20 min, judged by easy penetration of the potato center by a temperature probe. For the vacuum-seal boiling (VSBoil) group, whole unpeeled tubers were first sealed in vacuum bags by a vacuum sealer (Seal-a-Meal, Walmart, USA), then boiled in water for 20 min. Raw and cooked potatoes were then diced, freeze-dried (FreeZone 4.5, Labconco; Kansas City, MO, USA), powdered to an average particle size of about 0.2 mm, packaged, and stored at −80 °C until use.

The PP extract was prepared as previously described with modifications [10]. Briefly, powdered lyophilized PP was defatted twice with *n*-hexane (10:1; *v*/*w*, *n*-hexane: sample) at 70 °C for 20 min, followed by 1000× *g* centrifugation. Residues were extracted twice with 80% ethanol containing 1% formic acid (10:1; *v*/*w*, *n*-hexane: sample) on a rotary shaker (VWR, Radnor, PA, USA) at room temperature for 12 h. After centrifuging at 1000× *g*, the supernatant was collected and diluted to a final volume of 25 mL and stored at −80 °C until use. For the extracts used for cell culture, the supernatant was concentrated under a nitrogen evaporator (ThermoFisher, Waltham, MA, USA) and freeze-dried (FreeZone 4.5, Labconco).

### 2.3. Determination of Total Polyphenolic Compounds

Total polyphenolic content (TPC) was tested using Folin–Ciocalteu assay in a 96-well plate as described previously with modifications [12]. First, 12.5 μL of Folin–Ciocalteu reagent and 37.5 μL of 10% Na_2_CO_3_ were added to 0.2 mL of diluted extract. The mixture was then incubated at room temperature for 2 h. The absorbance was read at 760 nm using Synergy H1 microplate reader (BioTek, Winooski, VT, USA). GA and CGA were used for establishing calibration curves. The concentration of PP extract was expressed as mg GA equivalent (GAeq) per g dry weight (DW) of lyophilized PP powder or mg CGA equivalent (CGAeq) per g DW. Samples were analyzed in triplicate.

### 2.4. Quantification of CGA by HPLC

CGA content was quantified as described previously with modifications [13]. PP extracts were filtered through 0.45 μm filters before analysis. The analysis was performed by a high-performance liquid chromatography (HPLC) system (Shimadzu, Japan) equipped with CBM-20A communications bus modules, a DGU-20A degasser, a SIL-20AC auto-sampler, LC-20AD UFLC binary gradient pumps, a CTO-20A column oven, and a SPD-M30A diode array detector (190–600 nm). The sample separation was performed using a Waters (Milford, MA, USA) Resolve C18 column (3.9 mm × 150 mm; 5 µm particle size) at 30 °C The mobile phase consisted of 0.1% formic acid both in water (eluent A) and in methanol (eluent B). The gradient elution was at the flow rate of 0.5 mL/min with the following program: the initial ratio of B was 5% and kept for 2 min, raised to 25% at 26 min, increased to 90% at 27 min and maintained for 3 min, and then returned to 5% in 0.5 min and maintained for 7.5 min. CGA was used as the reference standard for quantitative analysis by peak areas. Their contents in different PP samples were calculated using an external standard method with corresponding calibration curves by peak areas. All samples were analyzed in triplicate.

### 2.5. DPPH Radical Scavenging Assay

The total antioxidant activity in PP extracts was determined by the ability to scavenge DPPH [14]. Briefly, 200 μL of DPPH solution (60 μM) was added into microplate wells containing 50 μL of 1:500 diluted PP extracts or CGA standard solution. The DPPH scavenging activity was measured at 515 nm after 90 min of incubation at room temperature. Data were expressed as mg CGAeq/g DW, which was calculated by the standard curve of CGA in the range of 0 to 10 μg/mL. The percent inhibition of DPPH radical scavenging activity was calculated by the following equation:

(1) $$\mathrm{inhibition}\left(\%\right)=\frac{A_{\mathrm{control}}{-A}_{\mathrm{sample}}}{A_{\mathrm{control}}}\;\times \;100$$

where *A* is the absorbance. Samples were analyzed in triplicate. After adding DPPH solution, the absorbance at 515 nm at 90 min was used to calculate the antioxidant activity of the extract with CGA standards (R^2^ = 0.995).

### 2.6. Cell Viability Assay

The lyophilized extracts were redissolved in 50% ethanol solution to 10 mg/mL, filtered through 0.2 μm filters before use, and the concentration of PP extracts was expressed as μg CGAeq/mL. Cell viability was tested by MTT assay, as described previously with modifications [10]. Caco-2 cells were seeded into each well in 96-well plates. After treatment with 0–100 μg CGAeq/mL PP extracts for 48 h, cells were incubated with 5 mg/mL MTT for 4 h. Formazan was resuspended in 100 μL DMSO, and the absorbance at 540 nm was measured using Synergy H1 microplate reader (BioTek, Winooski, VT, USA).

### 2.7. Intracellular Reactive Oxygen Species Assay

Intracellular reactive oxygen species (ROS) levels were measured using a cell-permeable fluorescent probe, DCFH-DA, as described previously with modifications [15]. Caco-2 cells were seeded into wells in a 96-well plate and cultured in DMEM complete media for 12 h. Then, cells were treated with 0.5% ethanol or 50 μg CGAeq/mL PP extract in DMEM complete media for another 12 h. Cells were washed with phenol red-free DMEM (Gibco, Waltham, USA) once and incubated with 100 μL of 10 μM fresh DCFH-DA for 45 min. After incubation, cells were washed with phenol red-free DMEM and then incubated with 100 μL of phenol red-free DMEM or 1 mM H_2_O_2_ for 30 min. Fluorescence of DCFH-DA in each well was assessed every 2 min within 30 min using a fluorescence plate reader (BioTek, Winooski, VT, USA) at an excitation wavelength of 485 nm and an emission wavelength of 530 nm.

### 2.8. Immunoblotting

Caco-2 cells were seeded into each well in a 12-well plate and cultured in DMEM complete media for 12 h. Then cells were treated with 5 ng/mL TNF-α with 0.5% ethanol or 50 μg CGAeq/mL PP extract for 24 h. Immunoblotting analysis was conducted according to procedures previously described [16]. Antibodies against phosphorylated p65 (phospho-p65) and p65 were purchased from Cell Signaling Technology (Beverly, MA, USA). Anti-β-tubulin antibody was obtained from DSHB (Iowa City, IA, USA). The binding of antibodies was detected using enzyme horseradish peroxidase coupled anti-rabbit or anti-mouse immunoglobulin and visualized using chemiluminescence. The density of bands was quantified and then normalized referencing the β-tubulin content.

### 2.9. Statistical Analysis

Statistical analyses were conducted using GraphPad Prism 7. Data were presented as mean ± standard error of the mean (SEM), and statistical difference was evaluated using student’s t-test and one-way ANOVA with Tukey post hoc. Significance was considered as *p*-value less than 0.05.

## 3. Results

### 3.1. Effect of Cooking Methods on Polyphenolic Content in PP

The extractable TPC of PP after boiling, VSBoil, or steaming was compared to that of raw PP by using the Folin–Ciocalteu assay, where GA and CGA were used as equivalent standards. Steam and Boil treatments reduced the TPC in PP compared to that in raw PP (Table 1). VSBoil retained a similar TPC as that in uncooked PP (Table 1).

**Figure 1:**
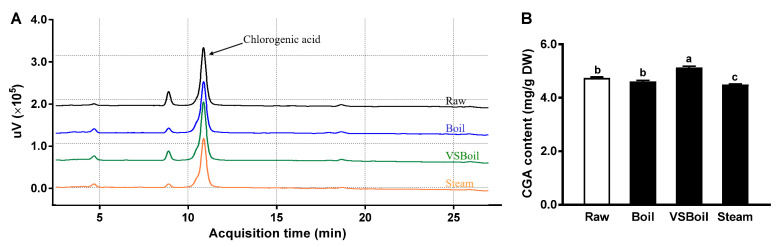
Chlorogenic acid content in PP extracts. (**A**). Representative high-performance liquid chromatography graphs of PP extracts at 320 nm. (**B**). Chlorogenic acid (CGA) content in PP extracts. DW: dry weight; VSBoil: vacuum-seal boiling. ^a–c^ means among bars without common letters differ significantly at *p* < 0.05 (mean ± SEM, *n* = 3).

The HPLC chromatograms of extracts of raw PP and PP after boiling, vacuum-seal boiling, and steaming at 320 nm are shown in Figure 1A, which had a retention time of CGA of 10.9 min. Using a CGA standard calibration curve (R^2^ = 0.9999), the CGA content in PP extracts was further quantified. The extractable CGA content in PP was deceased by steaming (by 5.4%) but was increased in the VSBoil group (by 8.2%; Figure 1B).

### 3.2. Effect of Cooking Methods on DPPH Radical Scavenging Activity

**Figure 2:**
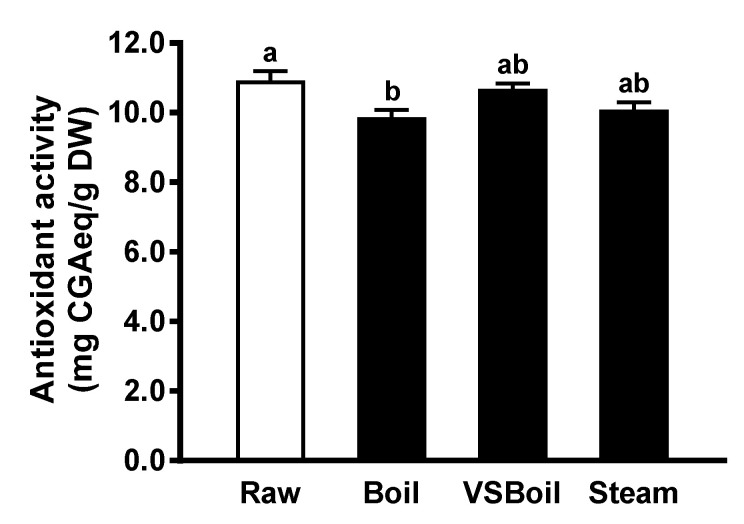
Free radical scavenging activity of raw and cooked purple potato (PP) extract. DW: dry weight; VSBoil: vacuum-seal boiling. ^a,b^ means among bars without common letters differ significantly at *p* < 0.05 (mean ± SEM, *n* = 3).

The antioxidant activity of PP extract from cooked PP was compared to that from raw PP by the DPPH method. As shown in Figure 2, regardless of the method, cooking reduced in vitro antioxidant activity of PP extracts. Polyphenol content extracted from boiled PP was significantly lower than that from uncooked PP (*p* < 0.05; Figure 2). The predicted concentrations for PP extracts from raw, boil, VSBoil, and steam groups to achieve 100% DPPH inhibition were 0.66, 0.73, 0.67, and 0.71 mg DW/mL, respectively.

### 3.3. Protective Effect of Extracts on Caco-2 Cell Viability and Intracellular ROS Production

**Figure 3:**
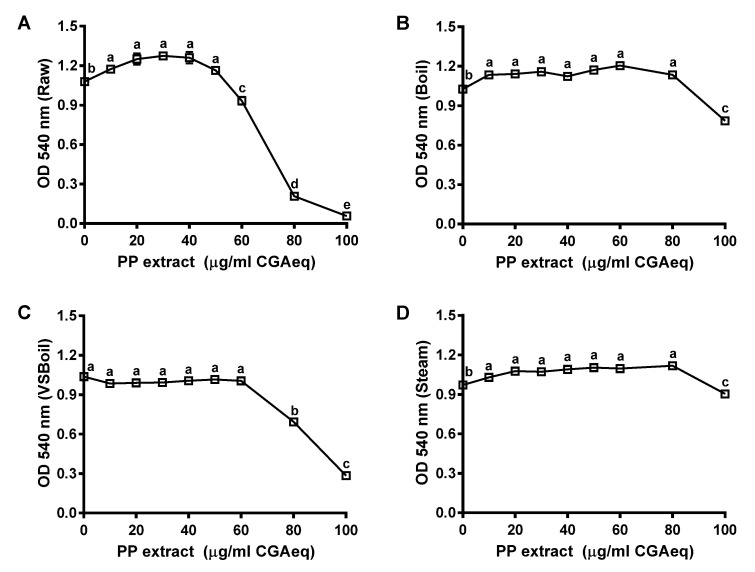
The cell viability of Caco-2 cell treated with raw and cooked purple potato (PP) extract. (**A**) Raw PP extract; (**B**) Boil; (**C**) VSBoil (vacuum-seal boiling); (**D**) Steam. ^a–e^ means among bars without common letters differ significantly at *p* < 0.05 (mean ± SEM, *n* = 6).

To assess the protective ability of PP extracts on intracellular ROS, the maximal dose of PP extracts without toxic effects on Caco-2 cells was evaluated first. As shown in Figure 3, extracts from raw, boiled, steamed, and VSBoiled PP maintained cell viability at concentrations up to 50, 80, 60, and 80 μg CGAeq/mL, respectively. Thus, a dose of 50 μg CGAeq/mL was selected for further cell-based tests.

**Figure 4:**
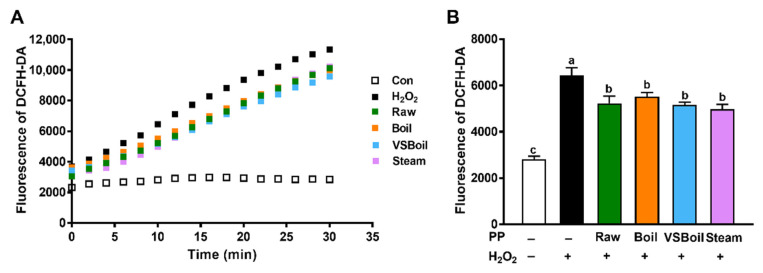
Effects of purple potato (PP) extracts on intracellular reactive oxygen species (ROS) production in human colonic Caco-2 cells induced by H_2_O_2_. (**A**) Intracellular ROS production was detected with DCFH-DA in Caco-2 cells pre-treated with PP extracts and followed by H_2_O_2_ induction for 30 min. (**B**) Intracellular ROS production in Caco-2 cells treated with PP extracts and H_2_O_2_ at 10 min. VSBoil: Vacuum-seal boiling. ^a–c^ means among bars without common letters differ significantly at *p* < 0.05 (mean ± SEM, *n* = 8).

ROS production within 30 min is shown as fluorescence intensity in Figure 4A. Caco-2 cells generated a ROS burst in response to H_2_O_2_ induction within 30 min, which was quenched effectively by all PP extract treatments (Figure 4A,B). Each extract had a similar degree of quenching effect (Figure 4B).

### 3.4. Anti-Inflammatory Effect of PP Extracts in TNF-α Treated Caco-2 Cells

**Figure 5:**
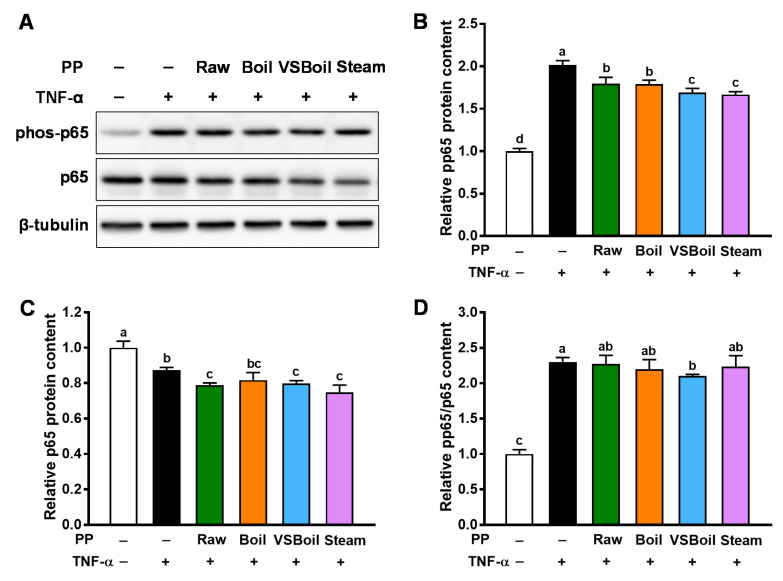
Anti-inflammatory effects of purple potato (PP) extracts on human colonic Caco-2 cells treated with tumor necrosis factor (TNF)-α. (**A**) Representative immunoblotting bands; (**B**) phospho-p65; (**C**) total p65; (**D**) ratio of phospho-p65 to p65. VSBoil: vacuum-seal boiling. ^a–c^ means among bars without common letters differ significantly at *p* < 0.05 (mean ± SEM, *n* = 4).

TNF-α is known to induce inflammation in intestinal epithelial cells [17,18,19]. Consistently, a 24 h TNF-α treatment at 5 ng/mL resulted in inflammation in Caco-2 cells as shown by enhanced phosphorylation of p65 and reduced total p65 protein content (Figure 5). PP extract supplementation, regardless of cooking method, reduced phosphorylation of p65 in Caco-2 cells (Figure 5A,B), indicating their anti-inflammatory activity. The extracts from raw, VSBoiled, and steamed PP further reduced the content of p65 in cells (Figure 5A–C). Our data show that, like raw PP extract, extracts from cooked PP can effectively suppress the inflammation in gut epithelial cells induced by TNF-α, with the VSBoil method being the most effective.

## 4. Discussion

The polyphenol content of raw Purple Pelisse PP used in this study was similar to that of the Vitelotte PP variety [20] but lower than that of the Heimeiren PP variety [11], which may be due to variation among cultivars and/or agricultural practices. TPC of Purple Pelisse PP in this study was similar to a previous report, where TPC was 7 mg/g GAeq DW, but the CGA content was higher than that in the previous study (2.44 mg/g DW) [21]. The observed difference could be due to agricultural practices. High-temperature cooking leads to the partial loss of polyphenolic compounds and TPC is strongly correlated with the in vitro antioxidant capacity of the extract [11,22,23,24]. Our data showed that raw PP extract had the highest TPC among the four extracts and the highest free radical scavenging activities. Polyphenolic compounds in PP are more concentrated in the peel than in flesh [25]. In this study, boiling and steaming significantly reduced TPC in PP, which might be due to oxidations induced by high temperatures and/or PP leaching during cooking. Consistently, boiling and steaming reduced the TPC and anthocyanin in Heimeiren PP, with the exception that steaming increased the CGA content [11]. However, boiling and steaming increased TPC, CGAs, and rutin in developmentally young potato tubers [26]. The solubility of CGA and other polyphenolic compounds in hot water and their surface distribution in potatoes may contribute to their loss after boiling and steaming. During cooking, we observed that water used to boil PP turned slightly blue, indicating the loss of anthocyanins and other colorless polyphenolic compounds. A vacuum-sealed bag can effectively prevent water contact but still transfer heat. VSBoil at 78–92 °C effectively avoided the leaching of anthocyanins [27], which might explain the higher retention of TPC and CGA in the VSBoil group. Besides, high temperature may also enhance the extraction of polyphenols by changing the cell wall structure. In support of our finding, boiling altered the cell wall structure and promoted the solubilization of pectic polysaccharides [28]. Grape cell wall polysaccharide deconstruction during fermentation is positively correlated with the grape polyphenolic content of wine [29]. The cooking induced deconstruction of the cellular structure, which, together with the isolation of the vacuum-seal bag, might have increased the polyphenol extractability, leading to a higher CGA content in VSBoil than raw PP. Inactivation of degradative enzymes through heating might have also contributed to the increased CGA in VSBoil compared to that in raw PP [25,26], which should be a minor factor considering that the CGA content was decreased in Boil and Steam groups compared to that in raw PP.

The polyphenol-rich extracts from PP showed free radical scavenging activity, ameliorated H_2_O_2_-induced ROS production in Caco-2 cells, and reduced inflammatory signaling in Caco-2 cells in response to TNF-α treatment. Consistently, CGA, a main polyphenolic compound in white potato, inhibited TNFα- and H_2_O_2_-induced interleukin-8 production in Caco-2 cells [30] and decreased H_2_O_2_ and mitochondrial superoxide overproduction in osteoblastic cells [31]. Dietary CGA supplementation also inhibited the mucosal damage caused by high-fat diet intake in rats [32] and increased duodenal glutathione peroxidase and catalase activity in weaned piglets [33]. Similarly, delphinidin-3-rutinoside, an anthocyanin found in purple potato [34], decreased tert-butyl hydroperoxide-induced intracellular ROS and prevented the reduction of glutathione to oxidized glutathione ratio in MC3T3-E1 osteoblastic cells [35].

Although the effect of cooking method on the antioxidant capacity of PPs has been previously studied by chemical analyses such as DPPH assay, ferric reducing antioxidant power assay, and colorimetric radical scavenging assay [26,36,37], live cells provide better information due to their physiological relevance. This study assessed the protective effects of PP extracts using an intestinal epithelial cell model, Caco-2 cells. The cell viability test of the PP extracts on Caco-2 cells indicated that the PP extracts of the cooked potatoes, regardless of the cooking method, were less toxic compared to the PP extract from raw potato, which was likely due to the presence of toxic substances in the raw PP extract. Naturally occurring glycoalkaloids, α-chaconine and α-solanine, are two prevalent toxicants in potatoes [38]. Peeling and cooking reduces these toxicants [39]. Cooking such as steaming and boiling after peeling can further reduce the content of α-solanine, α-chaconine, and total glycoalkaloids [40]. Using human colonic epithelial cells, we showed that the same concentration of PP extracts from raw or cooked PP, regardless of cooking methods, exhibited similar antioxidant and anti-inflammatory effects, indicating that the cooked potato retained the bioactivity and functionality of polyphenolic compounds in live cells. In support of our finding, boiling and baking increased recoverable amounts of polyphenols including CGAs, rutin, and kaempferol 3-O-rutinoside in baby PP [26]. Dietary baked PP supplementation attenuated dextran sodium sulfate (DSS)-induced colitis and associated gut inflammation in mice [41]. In addition, cooking processing might improve the antioxidant properties of naturally occurring antioxidants through modification and formation of the new compounds [42].

## 5. Conclusions

PP extracts from raw or cooked PP exhibited antioxidant and anti-inflammatory activities. The cooking process, regardless of method, maintained most of the polyphenol content and bioactivities in PP. Compared to the boil and steam groups, the VSBoil group retained a higher amount of TPC and extractable CGA, and had the highest antioxidant and anti-inflammatory activities. Cooked PP retained its protective effects against oxidative and inflammatory stress, with VSBoil being the preferred cooking method for maximizing extractable polyphenol content and beneficial effects. Though VSBoil cooking was highlighted in this study, more studies based on gastrointestinal digestion and human consumption are needed to make a solid conclusion.

## Figures and Tables

**Table 1:** Total polyphenolic content in purple potato after different cooking treatments.

	Linear Range (μg/mL)	R^2^	Content in Purple Potato (mg/g Dry Weight)			
			Raw	Boil	VSBoil	Steam
CGAeq	0–51.2	0.991	14.75 ± 0.30 ^a^	13.53 ± 0.05 ^bc^	14.47 ± 0.58 ^ab^	11.45 ± 0.19 ^d^
GAeq	0–25.6	0.994	6.50 ± 0.11 ^a^	6.00 ± 0.01 ^bc^	6.38 ± 0.23 ^ab^	5.19 ± 0.07 ^d^

CGAeq: chlorogenic acid equivalent; GAeq: gallic acid equivalent. VSBoil: vacuum-seal boiling. ^a–d^ mean of TPC within a row without a common letter differ significantly at *p* < 0.05 (mean ± SEM, *n* = 3).

## Data Availability

Data is contained within the article.
